# Therapeutic Effects of Mercury in Acute Periostitis

**Published:** 1866-02

**Authors:** Henry S. Chase


### THERAPEUTIC EFFECTS OF MERCURY IN ACUTE PERIOSTITIS.

By Henry S. Chase, D.D.S., M.D.

In an essay on Dental Therapeutics, published in the Dental Register, No. 1 for 1865, I said that mercury is our great dental polychrest. I presume that few who read that article gave the subject any further attention, as I treated the whole matter from a homœopathic stand point. There were some who personally thanked me for having written as I did.

I should like now to direct the attention of my brethren to a few cases which I have treated in the last four months, in fact *all* the cases of active periostitis which I have treated in that time.

Case 1.—Miss G., aged 13, strumous diathesis. Has thirteen gold plugs, lately inserted (August). About a month after the operation, teeth became sore to touch; could not eat on either side of the mouth with any comfort. Teeth very sensitive to thermal changes. Had been kept awake at night with pain. This case was cured in a week, and was relieved in twelve hours, under the influence of *mercurius vivus;* one thousandth of a grain being taken once an hour. That is the 3d prep. of mer. viv. was taken once an hour; dose, one grain.

Case 2.—Mr. C. aged 35. Healthy habit; of the nervo-bilious type.

Left superior bicuspid plugged with Lawrence's Amalgam, after removal of pulps, and a week of subsequent treatment.

One week after plugging, and during a wet "spell" in October had periostitis. Suffered two days before calling for treatment.

To take *mercurius vivus, third decimal* preparation, one grain every hour, twelve hours, or until better. The next day the patient reported himself cured; no trouble to this date, Dec. 23d.

Case 3.—Mr. D., aged 21. Healthy habit, of nervous temperament. Had rachitis when a boy. Has lateral curvature of the spine. Left inferior third molar decayed nearly to pulp. Plugged with Lawrence's Amalgam, July, 1865. About two weeks after operation, called and said he had "taken no comfort with his teeth since they were filled." Tooth is now *sore* and *loose.* To take *mercurius vivus,* third decimal preparation, every hour, twelve hours, or until better. Dose, one grain. Saw patient again in three days. He said, "I am all right now; don't know whether it was the medicine or not."

No trouble since to this date.

Case 4.—Miss. McP., aged twelve. Nervo-lymphatic habit, teeth show effects of hereditary syphilis. Front teeth notched. Spots, on many of the teeth, of a dead yellow color; particularly near the cutting edges and grinding surfaces. A very thin pellicle over these spots is hard and glassy; but immediately under, not deeper than the thickness of No. 6 gold foil, the enamel is as soft as chalk. The peculiar condition of the teeth have no immediate connection with the pathology and treatment of this case, and is only mentioned because interesting.

About twenty cavities were plugged with gold in September, 1865. The left superior second bicuspid was plugged with Lawrence's Amalgam, because it was much broken, pulp nearly exposed, and was colored with the yellow spot before mentioned. December 10th, Patient called and complained of this tooth. Would not bear heat or cold. Was sore and loose. Thinks she caught cold skating. Lanced the gums. To take *mercurius vivus*, as in the other cases already mentioned. December 14. No better. In consideration of patient's age, and condition of other teeth, resolved to extract *this* tooth. An examination of it, after removal, showed the pulp nearly exposed near line of junction between enamel and cementum; also the *horns* of the pulp extending half way to the cusps of the tooth, and very thinly covered with dentine.

Case 5.—Mr. S., age 55, a worn out farmer, naturally robust, of sanguine temperament. August 11th. Plugged right upper second molar on anterior approximal surface with Lawrence's Amalgam.

Oct. 12. Patient called, having periostitis, of recent date. Tooth sore and loose. Scarified gums. To take six powders of *mercurius vivus;* one grain once an hour.

Oct. 14. Much relieved, but is very sensitive to thermal changes. I ought to have removed the plug to day but did not. To take mercury as before.

Nov. 3d. Patient called. Tooth still sore, but not sensitive to thermal changes. Says the medicine relieves him every time, but the tooth will not *stay* cured. Concluding the pulp to have died from inflammation, caused by thermal changes. I removed plug, and finding a dead pulp, removed it and treated as usual.

Nov. 10th. Tooth still sore; syringed it well. Take mercury, as before.

Nov. 17th. Mercury relieved it; is better. Plugged with tin foil.

Dec. 26th. Have not heard from my patient since last date.

Case 6.—Master S., age 11, nervous temperament. Suspected of improper sexual habits.

Dec. 11th. Had the right under first molar plugged with Lawrence's Amalgam. Tooth very much decayed, and pulp nearly exposed. A week previous had saturated with creosote, and plugged with plaster of Paris. Before introducing the Amalgam, had cut out three-fourths, or more, of the plaster.

Dec. 15th. Patient called to have the tooth extracted. Said he could not *skate* without making his tooth ache, as he had to keep his mouth open. Scraped the amalgam plug, so as to be sure it would not receive the pressure of the antagonizing tooth.

Dec. 22d. Patient called and insisted on extraction. I refused. To take twelve powders of *mercurius vivus*, one grain every hour until better.

Dec. 27th. Patient not having called again, suppose the case relieved.

Case 7.—Miss McN., age 28, sanguine temperament. Teeth nearly destroyed by decay. Dec. 23d. Wishes something to take for tooth ache. Pain principally located in the roots of left upper bicuspids, which were sore and loose. Refused to have them extracted at present, but very anxious for relief. To take *mercurius vivus*, as in other cases mentioned, and call again if not relieved. Dec. 28th. Patient has not called yet. Suppose case relieved.

Case 8.—Miss R., age 15. brunette. Weak constitution. Only child. Has always been indulged in eating, very much to the injury of her teeth. Plugged twenty-two cavities with gold.

Right under first molar had pulp removed, Oct. 2d. Plugged with gold Oct. 6th.

Dec. 1st. Patient called, complains of pain in last named tooth. Sore and loose. To take *mercurius vivus* as in other cases reported.

Dec. 28th. Have not seen patient since last date.

I do not treat *all* cases with mercury, for I occasionally have a patient who will not take it. To-day, December 28th, I had a case in which I used creosote and tannin in the pulp cavity, tinct. of aconite as topical lotion, and bled the gums. This is the only case which has refused mercury in several months.

